# Real-Time Localization of Magnetic Target Using Second-Order Scalar Magnetic Gradients

**DOI:** 10.3390/s26144402

**Published:** 2026-07-10

**Authors:** Nan Li, Menghui Qin, Leling Li, Liming Fan, Xiaoming Cao

**Affiliations:** 1 Navy Submarine Academy, Qingdao 266199, China; cxmdlmu@163.com; 2 School of Marine Science and Technology, Northwestern Polytechnical University, Xi’an 266200, China; qinmenghui@mail.nwpu.edu.cn (M.Q.); lileling@mail.nwpu.edu.cn (L.L.); limingfan@nwpu.edu.cn (L.F.)

**Keywords:** magnetic anomaly, target localization, second-order scalar magnetic gradient, CPA point

## Abstract

Magnetic anomaly generated by magnetic target is widely used in many areas. In this paper, a real-time magnetic target localization method based on second-order scalar magnetic gradients at the closest point of approach (CPA) is proposed. By exploiting the geometric symmetry of the magnetic anomaly field at the CPA point, closed-form expressions of the target position and magnetic moment are derived directly from the second-order spatial derivatives of scalar magnetic anomaly under the induced-magnetization assumption, thereby avoiding iterative global optimization. Furthermore, a residual-based error index is constructed to evaluate the consistency between measured and reconstructed second-order scalar magnetic gradients, enabling automatic determination of the CPA point during platform motion. The proposed method is validated by the experiment. The results show that the CPA point on the trajectory can be accurately identified using the proposed error index, and the localization accuracy is significantly improved near the CPA point. At the CPA point, the relative errors of the estimated distances and angles between the target and the two sensors are 0.89% and 0.38%, and 1.0% and 0.52%, respectively, while the relative error of the estimated magnetic moment magnitude is 4.85%. Therefore, the proposed method has great value in target localization based on a mobile magnetic anomaly detection system.

## 1. Introduction

The superposition of localized perturbations from ferromagnetic targets onto the ambient geomagnetic field causes spatial magnetic distortions, which is called magnetic anomaly. Owing to its strong penetration capability through seawater, soil, and vegetation, magnetic anomaly detection (MAD) has been widely applied in unexploded ordnance (UXO) [1,2], ferromagnetic pipelines [3,4], abandoned oil/gas well locations [5,6,7], and human medical investigation [8]. Compared with active sensing modalities such as sonar or radar, MAD systems exhibit advantages in covert operation, low energy consumption, and environmental adaptability, making them particularly attractive for mobile detection platforms including unmanned underwater vehicles [9], unmanned surface vehicles, and airborne magnetic survey systems [10,11].

To utilize magnetic anomaly information in these applications, accurate localization of magnetic targets is essential. The localization of magnetic targets is commonly established under the magnetic dipole approximation, where the target is represented by an equivalent dipole characterized by its spatial position and magnetic moment vector [12,13,14]. Existing magnetic localization methods can generally be divided into two categories: optimization-based methods and closed-form analytical methods. Optimization-based methods, including genetic algorithms, particle swarm optimization [15,16], population-based incremental learning, and Gauss–Newton iterative schemes, estimate target parameters by minimizing the discrepancy between measured and modeled magnetic responses. Although these methods exhibit strong adaptability, they usually involve nonlinear iterative solving, suffer from high computational complexity, and are vulnerable to local minima and initialization sensitivity, thereby limiting their real-time capability on mobile platforms. Closed-form analytical methods directly estimate the dipole position from magnetic field. Nara et al. [12] derived a closed-form formula using the magnetic field vector and its first-order spatial gradients. Fan et al. [17]. proposed a fast linear algorithm based on total magnetic field gradients. These methods provide high computational efficiency, but their performance strongly depends on accurate vector-field measurements and background-field compensation. To improve robustness, several studies introduced rotational invariants and tensor contraction quantities, leading to STAR-based localization methods and magnetic gradient contraction approaches [18]. Nevertheless, these methods are still affected by tensor measurement noise, asphericity errors, and finite-baseline approximation errors. More recently, multi-point MGT localization approaches have been proposed to improve localization observability by combining tensor measurements from multiple positions or multiple sensors [19]. While these methods improve localization stability, they generally require complicated array configurations, nonlinear optimization procedures, or strict geometric constraints among observation points.

In addition to algorithmic limitations, practical gradient-based localization systems are subject to systematic errors introduced by sensor array imperfections. In particular, uncertainties in sensor position and orientation within the array constitute a critical yet often overlooked error source: positional misregistration directly biases the finite-difference approximations of spatial gradient. Addressing such uncertainties requires rigorous array calibration. Simplex-based optimization has been applied to determine sensor geometry in magnetoencephalography (MEG) arrays [20], while co-registration error analysis frameworks have been established for OPM-based MEG systems to jointly quantify positional and orientational misalignment [21]. In electromagnetic measurement facilities, automated iterative algorithms have been developed to estimate magnetic sensor position and orientation under operational conditions [22]. The generality of sensor co-registration challenges in multi-sensor platforms is further illustrated by efficient optimization-based extrinsic calibration methods applied to heterogeneous sensor pairs [23]. These works collectively demonstrate that geometric calibration of the sensor array is a prerequisite for reliable gradient measurement. Such calibration substantially enhances the accuracy and robustness of gradient-based target localization.

Despite these advances, an important characteristic of mobile magnetic localization remains insufficiently exploited: the geometric structure of the sensor trajectory itself. In practical mobile MAD systems, the sensor platform continuously moves along a trajectory rather than remaining at a fixed observation point. Along this trajectory, there exists a unique closest point of approach (CPA) point, at which the distance between the moving sensor and the magnetic target reaches its minimum [24,25]. At the CPA point, the spatial distribution of the magnetic anomaly field exhibits a special geometric symmetry, leading to simplified analytical relationships between the target parameters and the spatial derivatives of the magnetic anomaly. However, most existing localization methods treat each measurement point independently and do not explicitly utilize this trajectory-dependent geometric constraint. Consequently, the localization estimates may fluctuate significantly during platform motion, and additional post-processing is usually required to determine the most reliable localization epoch.

To address these issues, this paper proposes a real-time magnetic-source localization method based on the second-order gradient of scalar magnetic anomaly at the CPA point. Different from conventional MGT methods that utilize the first-order gradient tensor of vector magnetic fields, the proposed approach derives localization relations directly from the second-order spatial derivatives of scalar magnetic anomaly measured by a moving scalar-gradient array. By exploiting the geometric constraint at the CPA point, closed-form expressions are established for the target position and magnetic moment under the induced-magnetization assumption. Furthermore, a residual-based error index is constructed to evaluate the consistency between the measured second-order scalar magnetic gradient and the reconstructed gradient generated by the estimated target parameters. The proposed error index provides a physically interpretable criterion for identifying the CPA point during platform motion, thereby enabling automatic selection of the optimal localization instant in real time.

The remainder of this paper is organized as follows. Section 2 derives the second-order gradient expressions of the magnetic anomaly based on the magnetic dipole model. Section 3 presents the closed-form localization formulas at the CPA point. Section 4 presents the real-time localization algorithm and the definition of the error index. Section 5 reports the experimental validation, and Section 6 concludes the paper.

## 2. Second-Order Gradient of Scalar Magnetic Anomaly Signal

**Figure 1:**
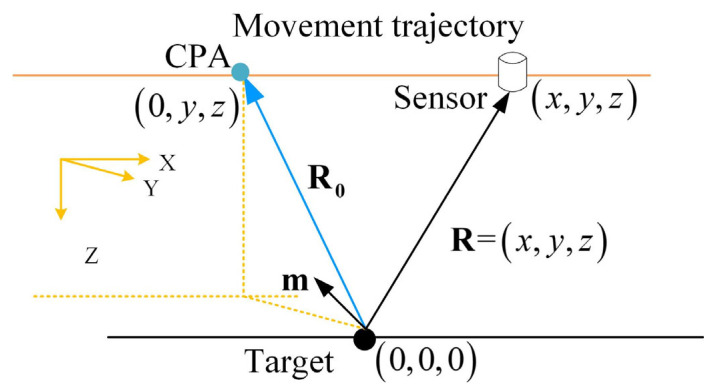
Model of magnetic anomaly detection.

A ferromagnetic target can be considered as a magnetic dipole when it is far enough away from the magnetic sensor [14]. The model of magnetic anomaly detection is shown in Figure 1, in which R represents the distance from the target to the sensor, and $m={\left[m_{x},m_{y},m_{z}\right]}^{T}$ represents the unit direction vector of the magnetic moment. The magnetic field generated by the dipole is expressed as:

(1) $$B_{a}=\left[\begin{array}{c} B_{ax}\\ B_{ay}\\ B_{az} \end{array}\right]=\frac{\mu_{0}M}{4\pi }\left(\frac{3(m\cdot R)R}{R^{5}}-\frac{m}{R^{3}}\right)$$

where $\mu_{0}=4\pi \times 10^{-7}H/m$ is the permeability of free space, and *M* is the magnitude of the magnetic moment of the dipole, R=|R|.

In practice, the total magnetic field detected by a magnetic sensor is the superposition of the ambient field $B_{e}$ and the field $B_{a}$ originating from the target. When a scalar magnetometer is employed to measure the magnetic anomaly ΔB caused by a distant target, this anomaly can be treated as the component of ***Bₐ*** projected along the direction of $B_{e}$, as [17]:

(2) $$\Delta B=\frac{B_{e}\cdot B_{a}}{\left|B_{e}\right|}=u\cdot B_{a}$$

where $u={\left[u_{x},u_{y},u_{z}\right]}^{T}$ is the unit direction vector of $B_{e}$.

To mitigate the effects of time-dependent changes in the ambient magnetic field, magnetic gradient measurements are commonly employed for anomaly detection. From Equation (2), the X-axis component of the gradient of ΔB is given by [17,21]:

(3) $$G_{x}=\frac{\alpha M}{R^{7}}(m\cdot R)\left[5(u\cdot R)x-u_{x}R^{2}\right]-\frac{\alpha M}{R^{5}}(u\cdot m)x-\frac{\alpha M}{R^{5}}(u\cdot R)m_{x}$$

where $\alpha =-\frac{3\mu_{0}}{4\pi }$.

From Formula (3), the second-order gradient of the scalar magnetic anomaly on the X-axis can be obtained as:

(4) $$G_{xx}=\alpha M\left(\begin{array}{c} 5m_{x}u_{x}\left(-7\frac{x^{4}}{R^{9}}+\frac{3x^{2}}{R^{7}}\right)+5\left(m_{x}u_{y}+m_{y}u_{x}\right)\left(-7\frac{x^{3}y}{R^{9}}+\frac{2xy}{R^{7}}\right)\\ +5\left(m_{x}u_{z}+m_{z}u_{x}\right)\left(-7\frac{x^{3}z}{R^{9}}+\frac{2xz}{R^{7}}\right)+5\left(m_{y}u_{z}+m_{z}u_{y}\right)\left(-7\frac{zyx^{2}}{R^{9}}+\frac{zy}{R^{7}}\right)\\ +5m_{y}u_{y}\left(-7\frac{y^{2}x^{2}}{R^{9}}+\frac{y^{2}}{R^{7}}\right)+5m_{z}u_{z}\left(-7\frac{z^{2}x^{2}}{R^{9}}+\frac{z^{2}}{R^{7}}\right)\\ -\left(3u_{x}m_{x}+u_{y}m_{y}+u_{z}m_{z}\right)\left(-5\frac{x^{2}}{R^{7}}+\frac{1}{R^{5}}\right)+5\left(u_{x}m_{y}+m_{x}u_{y}\right)\frac{xy}{R^{7}}\\ +5\left(u_{x}m_{z}+m_{x}u_{z}\right)\frac{xz}{R^{7}} \end{array}\right)$$

(5) $$G_{xy}=\alpha M\left(\begin{array}{c} 5m_{x}u_{x}\left(-7\frac{x^{3}y}{R^{9}}\right)+5\left(m_{x}u_{y}+m_{y}u_{x}\right)\left(\frac{x^{2}}{R^{7}}-7\frac{x^{2}y^{2}}{R^{9}}\right)\\ +5\left(m_{x}u_{z}+m_{z}u_{x}\right)\left(-7\frac{x^{2}zy}{R^{9}}\right)+5\left(m_{y}u_{z}+m_{z}u_{y}\right)\left(-7\frac{zy^{2}x}{R^{9}}+\frac{zx}{R^{7}}\right)\\ +5m_{y}u_{y}\left(\frac{2yx}{R^{7}}-7\frac{y^{3}x}{R^{9}}\right)+5m_{z}u_{z}\left(-7\frac{z^{2}xy}{R^{9}}\right)\\ +5\left(3u_{x}m_{x}+u_{y}m_{y}+u_{z}m_{z}\right)\frac{xy}{R^{7}}-\left(u_{x}m_{y}+m_{x}u_{y}\right)\left(\frac{1}{R^{5}}-5\frac{y^{2}}{R^{7}}\right)\\ +5\left(u_{x}m_{z}+m_{x}u_{z}\right)\frac{yz}{R^{7}} \end{array}\right)$$

(6) $$G_{xz}=\alpha M\left(\begin{array}{c} 5m_{x}u_{x}\left(-7\frac{x^{3}z}{R^{9}}\right)+5\left(m_{x}u_{y}+m_{y}u_{x}\right)\left(-7\frac{x^{2}zy}{R^{9}}\right)\\ +5\left(m_{x}u_{z}+m_{z}u_{x}\right)\left(\frac{x^{2}}{R^{7}}-7\frac{x^{2}z^{2}}{R^{9}}\right)+5m_{z}u_{z}\left(\frac{2zx}{R^{7}}-7\frac{z^{3}x}{R^{9}}\right)\\ +5\left(m_{y}u_{z}+m_{z}u_{y}\right)\left(-7\frac{z^{2}yx}{R^{9}}+\frac{xy}{R^{7}}\right)+5m_{y}u_{y}\left(-7\frac{zxy^{2}}{R^{9}}\right)\\ +5\left(3u_{x}m_{x}+u_{y}m_{y}+u_{z}m_{z}\right)\frac{xz}{R^{7}}+5\left(u_{x}m_{y}+m_{x}u_{y}\right)\frac{yz}{R^{7}}\\ -\left(u_{x}m_{z}+m_{x}u_{z}\right)\left(\frac{1}{R^{5}}-5\frac{z^{2}}{R^{7}}\right) \end{array}\right)$$

## 3. Magnetic Target Localization at CPA Using Second-Order Gradient

In the magnetic anomaly detection setup illustrated in Figure 1, the magnetic sensor travels at a constant velocity along a straight path that lies in the XOY plane and is parallel to the X-axis. A particular point on this trajectory, referred to as the CPA (closest point of approach), marks the location where the target is nearest to the sensor’s path. With respect to the coordinate system shown in Figure 1, the CPA is located at coordinates (0, y, z). At this CPA position, the second-order gradient of the scalar magnetic anomaly caused by the target can be reduced to the simplified form presented below:

(7) $$\{\begin{array}{c} G_{xx}=\alpha M\left(\left(4m_{y}u_{y}-3u_{x}m_{x}-u_{z}m_{z}\right)\frac{y^{2}}{R^{7}}+5\left(m_{y}u_{z}+m_{z}u_{y}\right)\frac{zy}{R^{7}}+\left(4u_{z}m_{z}-3u_{x}m_{x}-u_{y}m_{y}\right)\frac{z^{2}}{R^{7}}\right)\\ G_{xy}=\alpha M\left(4\left(u_{x}m_{y}+m_{x}u_{y}\right)\frac{y^{2}}{R^{7}}+5\left(u_{x}m_{z}+m_{x}u_{z}\right)\frac{yz}{R^{7}}-\left(u_{x}m_{y}+m_{x}u_{y}\right)\frac{z^{2}}{R^{7}}\right)\\ G_{xz}=\alpha M\left(-\left(u_{x}m_{z}+m_{x}u_{z}\right)\frac{y^{2}}{R^{5}}+5\left(u_{x}m_{y}+m_{x}u_{y}\right)\frac{yz}{R^{7}}+4\left(u_{x}m_{z}+m_{x}u_{z}\right)\frac{z^{2}}{R^{7}}\right) \end{array}$$

where $R=\sqrt{y^{2}+z^{2}}$.

In general, the magnetic moment of the target consists of two parts: intrinsic magnetic moment and induced magnetic moment [26]. For some targets, their intrinsic magnetic moment is artificially eliminated on the special purpose, or the magnitude of intrinsic magnetic moment is much less than that of induced magnetic moment. Therefore, for these targets, their magnetic moment is dominated by the induced component. However, the direction of the induced magnetic moment is not necessarily parallel to the ambient geomagnetic field in all cases. For example, if the target has anisotropic magnetic susceptibility or a conductive response under an alternating electromagnetic field, the induced magnetic response may deviate from the direction of the external magnetic field.

In this study, the proposed closed-form localization formula is derived for a passive quasi-static magnetic anomaly scenario. The target is approximated as a single equivalent magnetic dipole, and the remanent magnetization is assumed to be negligible compared with the induced magnetization. Moreover, the effective magnetic susceptibility of the target is assumed to be approximately isotropic. Under these conditions, the induced magnetic moment can be approximated as being aligned with the local geomagnetic field. Therefore, the magnetic moment direction can be represented by the unit vector of the geomagnetic field, namely m=u [22].

Under this assumption, Equation (7) can be rewritten in matrix form as:

(8) $$\left[\begin{array}{c} G_{xx}\\ G_{xy}\\ G_{xz} \end{array}\right]=\alpha M\left[\begin{array}{ccc} 4u_{y}^{2}-3u_{x}^{2}-u_{z}^{2} & 10u_{y}u_{z} & 4u_{z}^{2}-3u_{x}^{2}-u_{y}^{2}\\ 8u_{x}u_{y} & 10u_{x}u_{z} & -2u_{x}u_{y}\\ -2u_{x}u_{z} & 10u_{x}u_{y} & 8u_{x}u_{z} \end{array}\right]\left[\begin{array}{c} \frac{y^{2}}{R^{7}}\\ \frac{yz}{R^{7}}\\ \frac{z^{2}}{R^{7}} \end{array}\right]$$

Let $A=\alpha M\left[\begin{array}{ccc} 4u_{y}^{2}-3u_{x}^{2}-u_{z}^{2} & 10u_{y}u_{z} & 4u_{z}^{2}-3u_{x}^{2}-u_{y}^{2}\\ 8u_{x}u_{y} & 10u_{x}u_{z} & -2u_{x}u_{y}\\ -2u_{x}u_{z} & 10u_{x}u_{y} & 8u_{x}u_{z} \end{array}\right]$.

Due to |A|≠0, the localization information of the target can be obtained by multiplying both sides of Equation (8) by the inverse matrix of ***A***, as:

(9) $$\left[\begin{array}{c} \frac{y^{2}}{R^{7}}\\ \frac{yz}{R^{7}}\\ \frac{z^{2}}{R^{7}} \end{array}\right]=\frac{1}{\alpha M}\left[\begin{array}{ccc} c_{11} & c_{12} & c_{13}\\ c_{21} & c_{22} & c_{23}\\ c_{31} & c_{32} & c_{33} \end{array}\right]\left[\begin{array}{c} G_{xx}\\ G_{xy}\\ G_{xz} \end{array}\right]$$

where $A^{-1}=\frac{1}{\alpha M}\left[\begin{array}{ccc} c_{11} & c_{12} & c_{13}\\ c_{21} & c_{22} & c_{23}\\ c_{31} & c_{32} & c_{33} \end{array}\right]$, and $c_{ij}$ is only related to the components of u which can be considered as known coefficient.

Based on Equation (9), we can obtain the following equation as:

(10) $$\{\begin{array}{c} \frac{y^{2}}{R^{7}}=\frac{1}{\alpha M}\left(c_{11}G_{xx}+c_{12}G_{xy}+c_{13}G_{xz}\right)\\ \frac{yz}{R^{7}}=\frac{1}{\alpha M}\left(c_{21}G_{xx}+c_{22}G_{xy}+c_{23}G_{xz}\right)\\ \frac{z^{2}}{R^{7}}=\frac{1}{\alpha M}\left(c_{31}G_{xx}+c_{32}G_{xy}+c_{33}G_{xz}\right) \end{array}$$

Therefore, we can obtain the angle and distance between the sensor and the target at the CPA point based on Equation (10), as:

(11) $$\{\begin{array}{c} \tan\theta =\frac{y}{z}=\frac{c_{11}G_{xx}+c_{12}G_{xy}+c_{13}G_{xz}}{c_{21}G_{xx}+c_{22}G_{xy}+c_{23}G_{xz}}\\ \frac{1}{R^{5}}=\frac{{(c}_{11}+c_{31})G_{xx}+\left(c_{12}+c_{32}\right)G_{xy}+{(c}_{13}+c_{31})G_{xz}}{\alpha M} \end{array}$$

**Figure 2:**
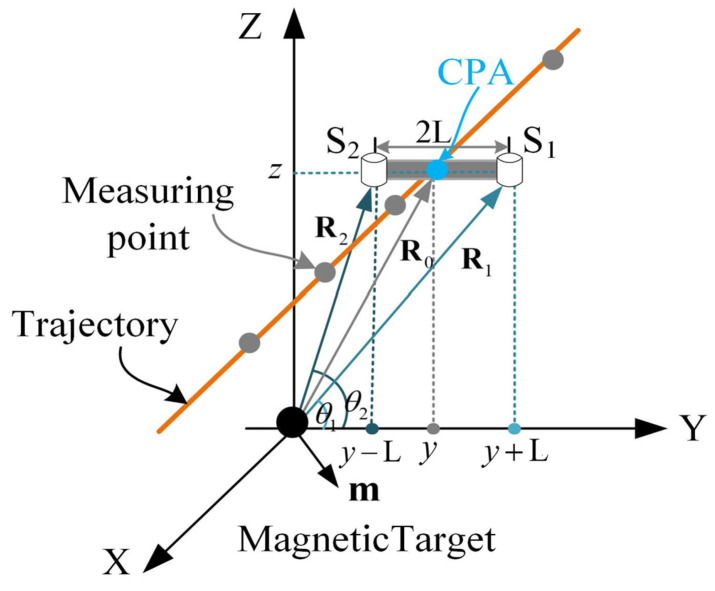
The localization of the target using the sensor array.

To determine the target’s position, a sensor array should be employed, as shown in Figure 2. The baseline with 2L of the array is arranged perpendicular to the trajectory. If we assume that the midpoint of this baseline lies on the trajectory, then when the array reaches the CPA point, we can derive the angles between the target and each of the two sensors:

(12) $$\{\begin{array}{c} \tan\theta_{1}=\frac{y+L}{z}=\frac{c_{11}G_{xx}^{S_{1}}+c_{12}G_{xy}^{S_{1}}+c_{13}G_{xz}^{S_{1}}}{c_{21}G_{xx}^{S_{1}}+c_{22}G_{xy}^{S_{1}}+c_{23}G_{xz}^{S_{1}}}\\ \tan\theta_{2}=\frac{y-L}{z}=\frac{c_{11}G_{xx}^{S_{2}}+c_{12}G_{xy}^{S_{2}}+c_{13}G_{xz}^{S_{2}}}{c_{21}G_{xx}^{S_{2}}+c_{22}G_{xy}^{S_{2}}+c_{23}G_{xz}^{S_{2}}} \end{array}$$

where $G_{xj}^{S_{k}}$ denotes the second-order scalar magnetic gradient measured by the *k*-th sensor.

From Equation (12), we can calculate the z position of the target at CPA, as:

(13) $$z=\frac{\tan\theta_{1}-\tan\theta_{2}}{2L}$$

Then, when the value of the z position has been calculated, the y position from the target to the corresponding sensor can be obtained based on Equations (10) and (11), as:

(14) $$y_{k}=z\tan\theta_{k}\pm L$$

Based on Equations (11), (13) and (14), we can calculate the magnitude of the magnetic moment M of the target using each sensor’s data, as:

(15) $$M=\frac{R_{k}^{5}}{\alpha }\left[\left(c_{11}+c_{31}\right)G_{xx}^{S_{k}}+\left(c_{12}+c_{32}\right)G_{xy}^{S_{k}}+\left(c_{13}+c_{31}\right)G_{xz}^{S_{k}}\right]$$

Finally, the position and magnetic moment of the target at the CPA point can be directly calculated based on the geometric relationship of the array.

In addition, the value of angle θ can be calculated using the different ratio in Equation (10). Then, multiple z position values can be calculated using different angles θ in Equation (13). Therefore, in order to improve the accuracy, we can obtain the mean value $\tilde{z}$ of the z position and calculate the values of the y position and magnetic moment of the target at the CAP point using $\tilde{z}$.

## 4. Localization Method Based on Second-Order Gradient Data

From Equations (12–15), we can obtain the parameters of the target at the CPA point. It means that the values of position and magnetic moment of the target are equal or close to the true values only that the sensor array moves to the CPA point. In other words, when the sensor array is far away from the CPA point, the calculated values of position and magnetic moment are inaccurate. Therefore, we define an error index in Equation (14) to evaluate whether the array is at the CPA point. The closer the array is moved to the CPA point, the better the estimate value of error index approximates 0.

(16) $$E=\frac{\sum_{k}{\left(G_{xj}^{S_{k}}-IG_{xj}^{S_{k}}\right)}^{2}}{\sum_{k}{\left(G_{xj}^{S_{k}}\right)}^{2}}\times 100,\;j=x,y,z$$

where $G_{xj}^{S_{k}}$ is the measured second-order scalar magnetic gradient by the *k*-th sensor. $IG_{xj}^{S_{k}}$ is the calculated second-order scalar magnetic gradient using the obtained parameters of the target at the CPA point.

**Figure 3:**
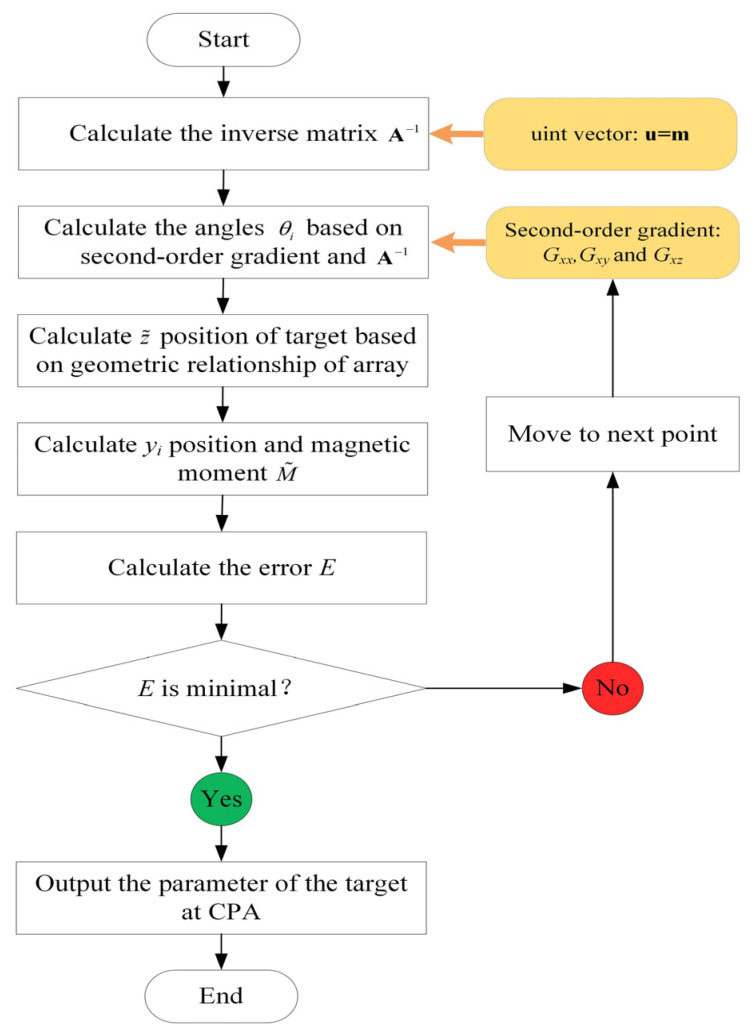
Flowchart of proposed method.

Based on Equation (16), the CPA point can be determined by the error index when its value is minimal, i.e., min(E). The calculated position and magnetic moment are the parameters of the magnetic target. Therefore, the target is located and its parameters are outputted. The flowchart of the proposed method for target localization using second-order scalar magnetic gradient is depicted in Figure 3, and the main steps of the method are summarized as follows.

Step 1: Treat the current measuring point as the CPA point and compute the target parameters using the second-order scalar magnetic gradient.

Step 2: With the obtained parameters, calculate the error index to assess whether the measuring point is indeed the CPA point.

Step 3: If the measuring point is confirmed as the CPA point, output the target parameters. If not, advance to the next measuring point and repeat Steps 1 and 2.

At every measuring point along the trajectory, the proposed method yields both the target parameters and the corresponding error index. By identifying the point where the error index attains its minimum, we can determine the target’s position and magnetic moment at the true CPA point.

## 5. Experiments

### 5.1. Experiment Design

#### 5.1.1. Second-Order Scalar Magnetic Gradient Measurement

**Figure 4:**
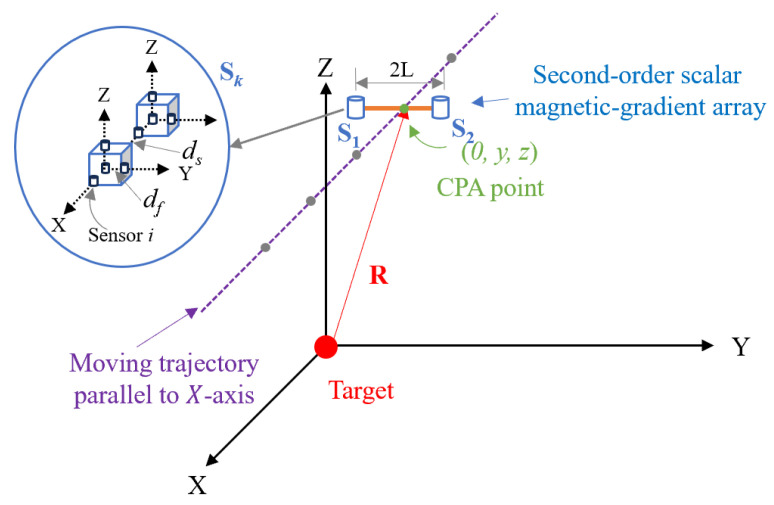
The configuration of the second-order scalar magnetic gradient measurement device.

To clarify how the second-order scalar magnetic gradient components used in the proposed localization method are obtained, the configuration of the gradient measurement device is illustrated in Figure 4. The whole localization array consists of two identical second-order scalar magnetic gradient measurement units, denoted by S_1_ and S_2_. The two units are separated by a localization baseline of 2L, and the center of the baseline moves along a straight trajectory parallel to the global *X*-axis. When the array center reaches the closest point of approach (CPA), the target is located at the relative position (0, y, z) with respect to the array-center coordinate system.

Each measurement unit S_k_ is composed of two first-order scalar-gradient sub-units separated along the local *x*-direction. Within each first-order sub-unit, scalar optically pumped magnetometers are arranged along the local *x*, *y*, and *z* axes. Since the optically pumped magnetometers measure scalar magnetic field rather than vector magnetic-field components, the first-order scalar-gradient components are obtained from the finite difference in the scalar magnetic anomaly ΔB. For a first-order sub-unit centered at **r**, the first-order scalar gradient along the *i*-direction is calculated as:

(17) $$G_{i}\left(r\right)=\frac{\partial \Delta B}{\partial i}\approx \frac{\Delta B\left(r+\frac{d_{s}}{2}e_{i}\right)-\Delta B\left(r-\frac{d_{s}}{2}e_{i}\right)}{d_{s}},\;\;i\in \{x,y,z\}$$

where $d_{s}$ is the first-order finite-difference baseline, and **e**_i_ is the corresponding unit vector.

The second-order scalar magnetic gradient components are then obtained by differencing the first-order gradient vector between the two sub-units separated along the local *x*-direction. Let $d_{f}$ denote the separation between the two first-order sub-units. The second-order scalar magnetic gradient can be approximated as:

(18) $$G_{ix}\left(r\right)=\frac{\partial G_{i}}{\partial x}\approx \frac{G_{i}\left(r+\frac{d_{f}}{2}e_{x}\right)-G_{i}\left(r-\frac{d_{f}}{2}e_{x}\right)}{d_{f}},\;\;i\in \{x,y,z\}$$

Thus, this finite-difference operation gives $G_{xx}$, $G_{yx}$ and $G_{zx}$. For a smooth scalar magnetic anomaly field, the Hessian matrix is symmetric, namely: $G_{yx}=G_{xy},\;G_{zx}=G_{xz}$.

Therefore, the required second-order scalar magnetic gradient components $G_{xx}$, $G_{xy}$ and $G_{xz}$ used in the CPA localization equations can be obtained from the scalar magnetic measurements. In the following localization procedure, $G_{xx}^{S_{k}}$, $G_{xy}^{S_{k}}$ and $G_{xz}^{S_{k}}$ represent the second-order scalar magnetic gradient components obtained by the *k*-th measurement unit $S_{k}$.

#### 5.1.2. Accuracy Evaluation of Second-Order Scalar Magnetic Gradient Measurement

The accuracy of the second-order scalar magnetic gradient measurement is mainly affected by the intrinsic noise of the scalar magnetometers, the finite-difference baselines, and the relative-position calibration of the sensors. Here, an error-propagation model is introduced to estimate the theoretical noise floor of the second-order scalar magnetic gradient components.

Assume that the noise of each scalar magnetometer is independent and has the same noise spectral density $n_{B}$. For the first-order finite difference in Equation (17), the noise spectral density of the first-order scalar-gradient component can be estimated as:

(19) $$n_{G_{i}}=\frac{\sqrt{n_{B}^{2}+n_{B}^{2}}}{d_{f}}=\frac{\sqrt{2}n_{B}}{d_{f}},\;\;\;i\in \{x,y,z\}$$

For the second-order finite difference in Equation (18), the first-order gradient noise is further propagated and amplified. The noise spectral density of the second-order component is

(20) $$n_{G_{ix}}=\frac{\sqrt{n_{G_{i}}^{2}+n_{G_{i}}^{2}}}{d_{s}}=\frac{\sqrt{2}n_{G_{i}}}{d_{s}}=\frac{2n_{B}}{d_{f}d_{s}},\;\;\;i\in \{x,y,z\}$$

In the experimental configuration, the intrinsic noise density of the CS-L optically pumped magnetometer is approximately $n_{B}=0.6\;\mathrm{pT}/\sqrt{\mathrm{Hz}}$ at 1 Hz. The first-order finite-difference baseline and the second-order finite-difference baseline are set as $d_{f}=1\;m$ and $d_{s}=1\;m$, respectively. According to Equation (18), the theoretical noise floor of the second-order scalar magnetic gradient measurement is approximately $1.2\;\mathrm{pT}\cdot m^{-2}/\sqrt{\mathrm{Hz}}@1\;\mathrm{Hz}$.

The main parameters of the second-order scalar magnetic gradient measurement are summarized in Table 1.

### 5.2. Theoretical Model Test Under Different Noise Levels

To further evaluate the localization accuracy and noise robustness of the proposed localization method, a controlled theoretical model test was conducted. The purpose of this test was to verify whether the closed-form localization equations and the residual-based CPA identification index remain effective under different second-order scalar magnetic gradient noise levels.

The theoretical model used the same target and trajectory parameters as those in the semi-physical experiment. The magnetic target was located at the origin of the coordinate system. The magnitude of the magnetic moment was set to M = 500 A·m^2^, and the inclination and declination of the magnetic moment were set to be the same as those of the local geomagnetic field, i.e., I = 63.3° and D = −10.1°. The localization baseline was 2L = 2 m. The array moved along the *X*-axis with a constant velocity from (−29.7, −12, 5) m to (29.7, −12, 5) m. Therefore, the theoretical CPA point was located at x = 0 m.

The theoretical second-order scalar magnetic gradient components $G_{xx}^{S_{k}}$, $G_{xy}^{S_{k}}$ and $G_{xz}^{S_{k}}$ of the two measurement units *S*_1_ and *S*_2_ were generated from the magnetic dipole model. To simulate different observation-noise conditions, zero-mean Gaussian noise was added to each second-order gradient component as:

$$G_{xj}^{S_{k}}=G_{xj}^{S_{k}}+\varepsilon_{xj}^{S_{k}},\;\;\;j=x,y,z,\;\;\;k=1,2$$

where $G_{xj}^{S_{k}}$ is the noisy second-order gradient component and $\varepsilon_{xj}^{S_{k}}$ is the additive gradient noise. The noise was assumed as $\varepsilon_{xj}^{S_{k}}\sim N(0,\sigma_{G}^{2})$, in which $\sigma_{G}=\eta n_{\nabla^{2}B}$.

The noise standard deviation was defined as

$$\sigma_{G}=\eta n_{\nabla^{2}B}$$

where $n_{\nabla^{2}B}=1.2\;\mathrm{pT}\;m^{-2}/\sqrt{\mathrm{Hz}}$ is the theoretical noise floor of the second-order scalar magnetic gradient measurement estimated in Section 5.1, and η is the noise-level coefficient. In this test, η = {0, 1, 2, 5, 10}, corresponding to $\sigma_{G}=\left\{0,1.2,2.4,6,12\right\}$, respectively. For each noise level, 100 Monte Carlo trials were performed.

First, the noise-free theoretical case was tested. The proposed method accurately identified the CPA point and recovered the target parameters. The estimated CPA position was x = 0.00 m, which was identical to the true CPA position. The estimated vertical position was z = 5.0000 m, and the estimated magnetic moment was M = 500.00 A·m^2^. This result verifies the correctness of the closed-form localization equations and the residual-based CPA identification criterion under ideal theoretical conditions.

The Monte Carlo results under different second-order gradient noise levels are summarized in Table 2. The results show that the localization error increases as the gradient noise level increases. When η = 1, corresponding to $\sigma_{G}$ = 1.2 pT·m^−2^, the CPA identification error was 0.03 ± 0.09 m, the distance error was 3.85 ± 2.84%, and the angle error was 0.21 ± 0.44°. When the noise level increased to η = 5, the CPA identification error increased to 0.34 ± 0.33 m, and the distance error increased to 6.42 ± 4.44%. Under the highest tested noise level, η = 10, the CPA error and distance error further increased to 0.50 ± 0.39 m and 11.54 ± 9.80%, respectively.

The Monte Carlo results indicate that the proposed method is sensitive to the noise level of the reconstructed second-order scalar magnetic gradients. Since these gradients are obtained through two finite-difference operations, scalar magnetometer noise is amplified during gradient reconstruction. Consequently, increasing environmental magnetic noise leads to larger CPA identification errors and parameter-estimation errors. As shown in Table 2, the method remains stable at the moderate noise level (η=1), whereas at (η=10) the CPA error increases to about 0.50 m and the range error exceeds 10%. The magnetic-moment estimate is the most noise-sensitive parameter because it depends directly on the gradient amplitude. In practical high-noise environments, temporal filtering, baseline optimization, and residual-index-based reliability checking should be used. If no distinct minimum of the residual index is observed, the localization result should be regarded as unreliable.

To evaluate the accuracy and computational efficiency of the proposed method, a comparison with PSO-based dipole fitting was added as an optimization-based baseline. The PSO method estimates the target parameters by minimizing the normalized residuals between the measured and modeled second-order scalar magnetic gradient components. Two simulation cases were considered: an ideal uniform straight-line trajectory and a trajectory-mismatch case in which the magnetic-gradient data were generated with a random velocity fluctuation within 10%. The localization results are shown in Table 3 and Table 4.

As shown in Table 3 and Table 4, the proposed method accurately identifies the CPA x-position in both trajectory conditions. Under the uniform straight-line trajectory, the proposed method gives a smaller position error than PSO, whereas PSO provides a more accurate magnetic moment estimate. Under the velocity-mismatch condition, the proposed method achieves lower position and range errors than PSO, indicating better robustness to moderate trajectory-model errors. More importantly, the proposed method requires only 10^−5^ s-level computation time, while PSO takes more than 1 s. Therefore, although the optimization-based method can improve some parameter estimates, especially the magnetic moment in the ideal case, the proposed method provides competitive localization accuracy with several orders of magnitude lower computational cost, which is more suitable for real-time CPA-based magnetic target localization.

### 5.3. Experimental Data and Results

#### 5.3.1. Data Acquisitions

To evaluate the localization algorithm under realistic magnetic-noise conditions while keeping the target parameters controllable, a semi-physical validation framework was adopted. In this framework, the measured environmental magnetic noise was superimposed on the analytical second-order scalar magnetic gradient signals generated by the magnetic dipole model.

**Figure 5:**
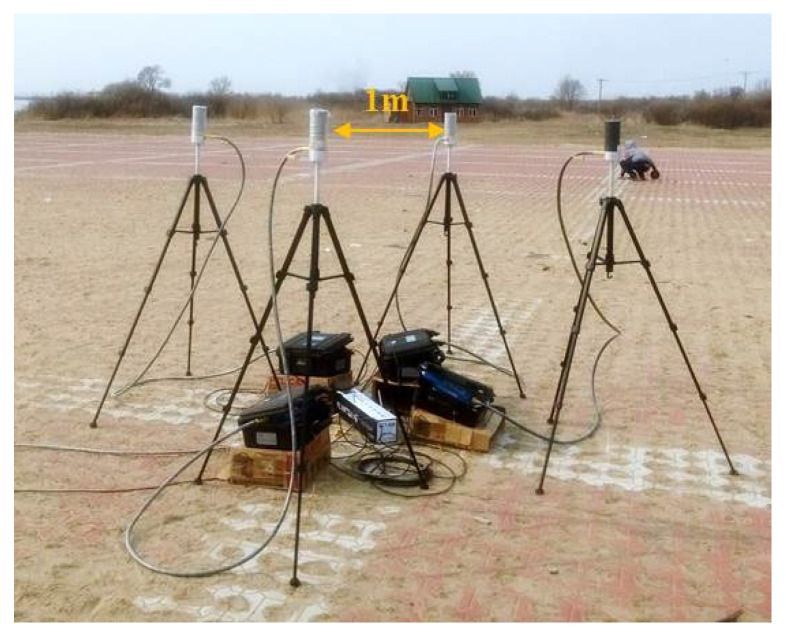
Sensor array with four magnetometers.

We acquired real magnetic noise gradient samples in Jinshatan Wetland Park in Harbin city, China, where the ambient magnetic activity was very low. The local inclination and declination of the geomagnetic field were 63.3° and −10.1°, respectively. In the experiment, we constructed a sensor array with four optical pumped magnetometers (CS-L, Scintrex) to measure the magnetic noise, as shown in Figure 5. The magnetometers were high-sensitivity, and intrinsic noise was about $0.6\;\mathrm{pT}/\sqrt{\mathrm{Hz}}@1\mathrm{Hz}$. The sample rate was 10 Hz.

**Figure 6:**
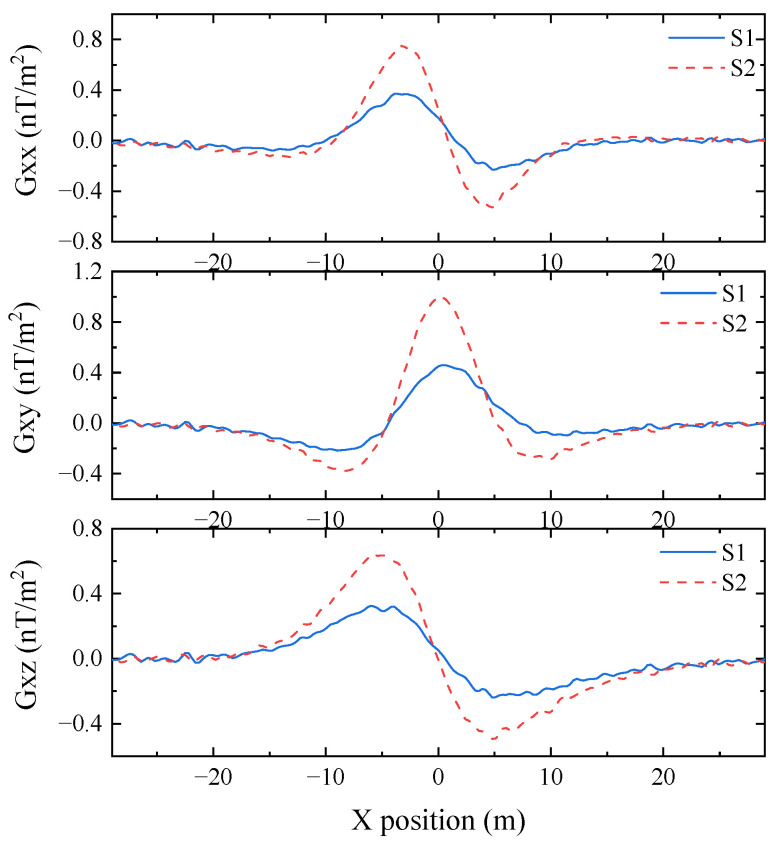
Second-order gradient of scalar magnetic anomaly.

The second-order gradient of the scalar magnetic anomaly was synthesized by the measured magnetic noise and the simulated magnetic anomaly. A typical second-order scalar magnetic gradient signal was acquired by the simulation. The target was located at the origin of coordinates. The magnitude of the magnetic moment M was set as $500\;A\cdot m^{2}$, and the inclination and deflection of target were set as that of the local geomagnetic field. The sensor array with the baseline of 2m moves along the X-axis with a constant velocity, starting from (−29.7, −12, 5) m and ending at (29.7, −12, 5) m. According to the above condition, the coordinate of the CPA point was (0, −12, 5) m. The synthesized second-order scalar magnetic gradient of two sensors with a segment of measured magnetic noise and the simulated magnetic anomaly is shown in Figure 6.

#### 5.3.2. Experiment Results

**Figure 7:**
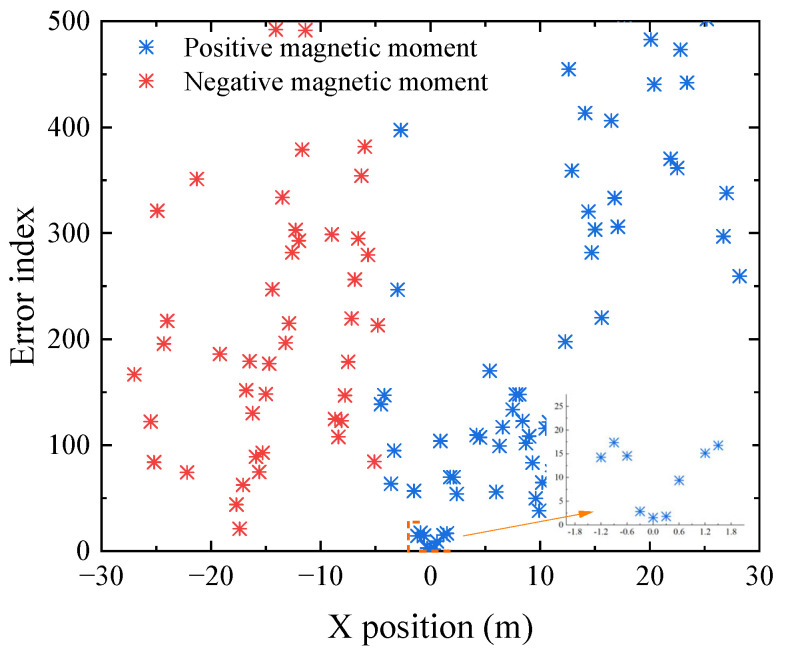
Error index of each point.

When the array moves on the trajectory, we can estimate the *y* position, *z* position and magnetic moment of the target at each measuring point using the second-order scalar magnetic gradient data. Then, based on the estimated parameters of the target, the error index can be calculated to determine whether the array reaches the CPA point. When the error index is at the minimal value, it means that the current measuring point is the CPA point on the trajectory. The parameters of the target calculated by the proposed method are considered as the components of the target. The error index of each point on the trajectory is shown in Figure 7. It can be seen that the error index is smaller when the points are close to the CPA point. When the array is at the CPA point (*x* = 0), the error index is 1.55, which is the minimal value. In Figure 6, the red points of the error index denote that the calculated magnitude of the magnetic moment is negative. It is known that the magnitude of the magnetic moment must be positive. It is obviously the wrong solution of the magnetic moment. Therefore, the CPA point is not one of these measuring points. The blue points of the error index denote that the calculated magnitude of the magnetic moment is positive. The minimal value of the error index in blue points should be the CPA point and the calculated *y* and *z* values at the corresponding point are the relative position between the sensor and the target.

To illustrate calculation results, we have chosen some measuring points with error indices less than 30 on the trajectory. The details of the calculation results are shown in Table 5 and the relative errors are shown in Table 6. It can be seen that the point with minimal error index is the CPA point on the trajectory. At the CPA point, the magnitude of the magnetic moment is obtained as $475.76\;A\cdot m^{2}$ and the relative error is 4.85%. The calculated distances $R=\sqrt{y^{2}+z^{2}}$ and angles between the target and sensor1 (S1) and sensor2 (S2) are 13.80 m, −68.69°and 11.96 m, −65.21°, respectively. The relative errors are 0.89%, 0.38% and 1.0%, 0.52%, respectively. The baseline $\left|y_{1}-y_{2}\right|$ of the array is 2 m based on the calculated parameters. It is consistent with the array structure. Therefore, the CPA point on the trajectory can be obtained and the parameters of the target at the CPA point can be calculated by the proposed method.

In addition, when the measuring points are close to the CPA point, the calculated parameters of the target are close to the true value. For example, at the measuring point with x position of 0.3 m, the magnitude of the magnetic moment is obtained as $574.09\;A\cdot m^{2}$ and the relative error is 14.81%. The relative error of distances and angles between the target and two sensors are 3.84%, 1.93% and 4.32%, 2.20%, respectively. Similarly, at the measuring point with x position of −0.3 m, the relative errors of the calculated parameters of the target are also smaller. Therefore, the parameters of the target calculated by the proposed approach are close to the true values at the measuring point which is near the CPA point.

In Table 5, it can be seen that the error indices of some measuring points are also small, which are far from the CPA point. For example, at the location where x = −17.4 x = −17.4 m, the error index is 21.06. Although the baseline length derived from the estimated parameters is 186.42 m, which does not strongly conflict with the array geometry, we can still readily recognize this as an incorrect solution.

The experimental results demonstrate that the proposed approach can estimate the target parameters in real time at the CPA point on the trajectory. The relative errors of the estimated parameters at the CPA point are relatively small. In addition, the CPA point itself can be located using the predefined error index.

## 6. Conclusions

In this paper, we concentrate on the real-time localization of magnetic targets and present an efficient method that utilizes the second-order scalar magnetic gradient of the anomaly. In this method, based on the characteristics of the second-order scalar magnetic gradient, we derive the expressions for both the position and the magnetic moment of the target at the CPA point along the trajectory, enabling the target parameters to be computed in real time. Additionally, we define an error index that serves to identify whether a given measurement point corresponds to the CPA point on the trajectory. The overall procedure is as follows: First, we assume that the current measuring point is the CPA point and compute the target parameters accordingly; then, using these parameters, we calculate the error index, and the CPA point is determined as the point on the trajectory that yields the minimum value of this error index. Finally, the position and magnetic moment of the target at the CPA point were outputted. The localization results showed that when the array was at the CPA point, the error of distances and angles between the target and two sensors were 0.89%, 0.38% and 1.0%, 0.52%, respectively. The error of magnitude of the magnetic moment was 4.85%. In addition, the error of parameters calculated by the proposed method was also smaller when the measuring points were near the CPA point.

It should be noted that the proposed closed-form formulation is most applicable to single-dipole targets whose induced magnetic moment can be approximated as aligned with the local geomagnetic field direction. For targets exhibiting strong remanent magnetization, anisotropic magnetic susceptibility, or frequency-dependent conductive responses, the magnetic moment direction should be estimated jointly with the positional parameters or calibrated independently prior to localization.

## Figures and Tables

**Table 1:** Parameters of second-order scalar magnetic gradient measurement.

Parameter	Symbol	Value
Magnetometer type	—	CS-L optically pumped scalar magnetometer
Number of magnetometers in the field experiment	—	4
Sampling rate	f_s_	10 Hz
Magnetometer noise density	n_B_	0.6 pT/√Hz @ 1 Hz
First-order finite-difference baseline	d_f_	1 m
Second-order finite-difference baseline	d_s_	1 m
Localization baseline	2L	2 m
First-order gradient noise floor	$n_{G_{i}}$	√2 n_B_/d_f_
Second-order gradient noise floor	$n_{G_{ix}}$	2 n_B_/(d_f_ d_s_)
Estimated second-order gradient noise floor	—	1.2 pT·m/√Hz @ 1 Hz

**Table 2:** Localization results under different noise levels.

η	*σ_G_*	CPA Error (m)	Distance Error (%)	Angle Error (Deg)	Magnetic Moment Error (%)	Valid Solution Rate (%)
0	0	0.00 ± 0.00	0.00 ± 0.00	0.00 ± 0.00	0.00 ± 0.00	100
1	1.2	0.03 ± 0.09	3.85 ± 2.84	0.22 ± 0.45	20.70 ± 17.28	100
2	2.4	0.14 ± 0.16	4.11 ± 2.88	0.82 ± 0.79	20.69 ± 16.19	100
5	6.0	0.35 ± 0.33	6.43 ± 4.44	2.03 ± 1.87	36.25± 30.26	100
10	12.0	0.50 ± 0.39	11.54 ± 9.80	3.08 ± 2.32	55.47 ± 54.70	100

**Table 3:** Localization under the uniform straight-line trajectory.

	CPA (m)			Magnetic Moment (A·m^2^)	Calculated Time (s)
	X-Position	Y-Position	Z-Position		
True value	0.00	−12.00	5.00	500.00	
Proposed method	0.00	−11.86	5.01	475.76	2.05 × 10^−5^
PSO method	0.01	−11.99	5.00	500.72	2.61

**Table 4:** Localization under the velocity-mismatch condition.

	CPA (m)			Magnetic Moment (A·m^2^)	Calculated Time (s)
	X-Position	Y-Position	Z-Position		
True value	0.00	−12.00	4.00	500.00	
Proposed method	0.00	−12.59	4.58	870.05	2.28 × 10^−5^
PSO method	0.08	−12.11	5.0537	522.6900	1.1755

**Table 5:** Calculation results of measuring points.

*X* Position (m)	Error Index	*y_1_* (S1) (m)	*y_2_* (S2) (m)	*z* Position (m)	*M* (Am^2^)
−17.4	21.06	−5.52	180.90	−14.36	−5.71 × 10^7^
−1.2	14.27	−12.02	−9.52	2.98	253.42
−0.9	17.39	−12.21	−9.55	3.32	279.70
−0.6	14.57	−11.87	−9.13	3.63	251.20
−0.3	2.87	−11.88	−9.79	4.23	298.41
0.0 (CPA)	1.55	−12.86	−10.86	5.01	475.76
0.3	1.81	−13.38	−11.34	5.50	574.09
0.6	9.41	−19.29	−16.65	8.68	3708.78
1.2	15.15	−16.01	−13.00	8.42	1252.98
1.5	16.78	−7.53	−5.44	4.01	22.49

**Table 6:** Relative error of measuring point.

*X* Position (m)	S1		S2		*M* (%)
	R_1_ (%)	Angle_1_ (%)	R_2_ (%)	Angle_2_ (%)	
−17.4	1120.291	23.92	752.47	25.02	1.10E7
−1.2	12.21	10.29	29.08	10.74	63.68
−0.9	9.15	8.48	16.35	8.07	49.31
−0.6	11.37	5.69	17.26	4.79	49.76
−0.3	9.46	−11.88	11.75	1.63	40.32
0.0 (CPA)	0.89	0.38	1.00	0.52	4.84
0.3	3.84	1.93	4.32	2.20	14.81
0.6	51.86	5.69	55.42	4.79	641.76
1.2	29.82	10.29	28.17	10.74	150.89
1.5	38.73	14.68	44.11	13.38	95.51

## Data Availability

Data are contained within the article.
